# A machine learning classifier reveals hidden rare biallelic heterozygous variants in the coding regions of Meniere disease genome

**DOI:** 10.1016/j.isci.2026.117838

**Published:** 2026-10-15

**Authors:** Sayedali A. Mohseni, Muhammad Hamza B. Khan, Pablo Cruz-Granados, Kiana Bagheri-Lotfabad, Amin Beheshti, Jose A. Lopez-Escamez

**Affiliations:** 1 Meniere Disease Neuroscience Research Program, Faculty of Medicine & Health, School of Medical Sciences, The Kolling Institute, University of Sydney, Sydney, NWS, Australia; 2 School of Computing, Centre for Applied Artificial Intelligence, Macquarie University, Sydney, NSW, Australia; 3 Otology & Neurotology Group CTS495, Division of Otolaryngology, Department of Surgery, Instituto de Investigación Biosanitaria, ibs.GRANADA, Universidad de Granada, Granada, Spain; 4 Ear Science Institute Australia, Nedlands, WA, Australia

**Keywords:** rare variant analysis, genomics, bioinformatics, vestibular disorders

## Abstract

Meniere disease (MD) is a rare inner ear disorder with a significant genetic contribution. We developed the MD classifier, an ML tool to distinguish familial from sporadic MD cases using rare variants in coding regions. We have generated a disease-specific gene variant score incorporating gene, variant, and disease-specific features. A gradient boosting classifier achieved the highest performance, with a test AUC of 0.843 (95% CI, 0.702–0.948) and an accuracy of 81.5%. Reclassification of sporadic cases into the familial group ranged from 16.6% to 28.3%. The model identified a burden of biallelic heterozygous variants suggesting hidden recessive inheritance that may affect up to 38% of MD patients. Gene-enrichment analysis using major classifier features revealed distinct biological pathways between familial and sporadic MD. This ML-based framework integrates unique genetic features that may improve the diagnostic yield of rare disorders and is universally applicable to conditions with both familial and sporadic phenotypes, enabling the identification of compound heterozygous recessive inheritance.

## Highlights

• A classifier distinguishes familial and sporadic MD using rare coding variants
• A burden of missense and synonymous variants in MD suggests hidden recessive inheritance
• The classifier is applicable to rare diseases with both familial and sporadic cases

## Graphical abstract

**Figure:**
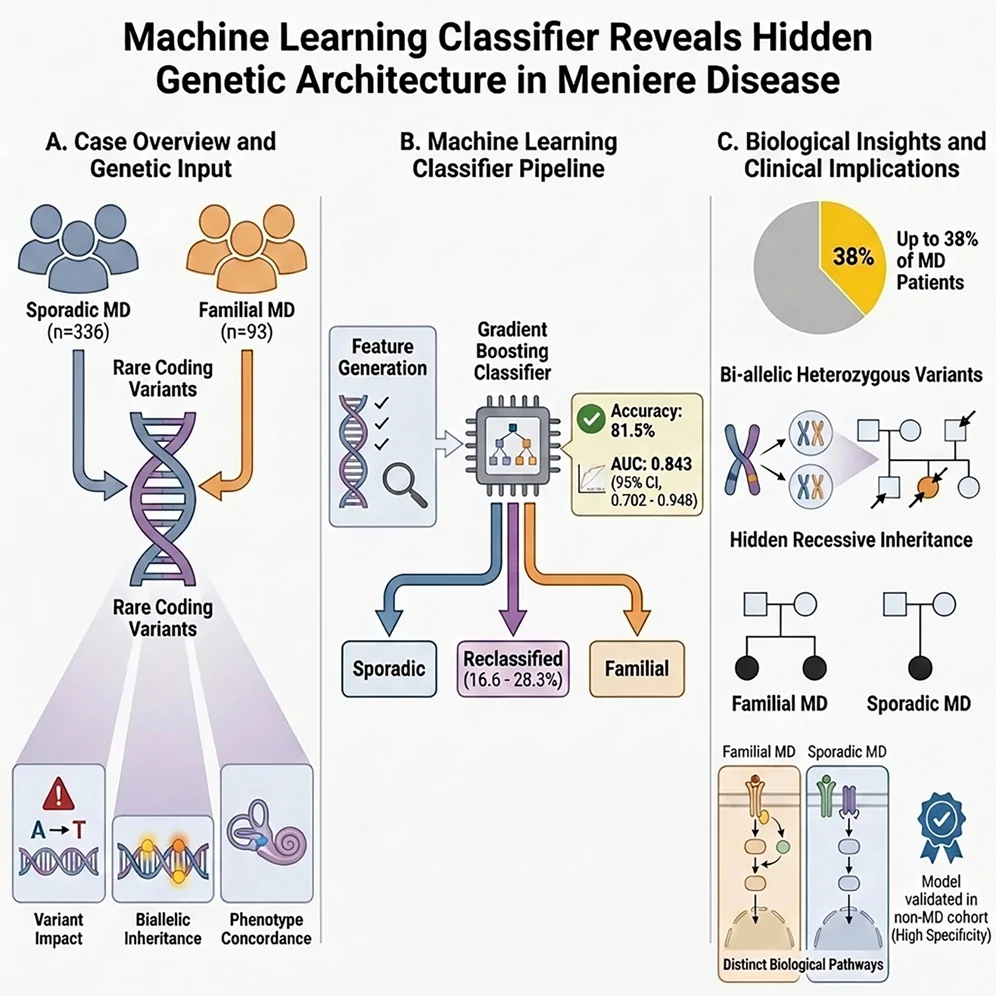

## Teaser

Health sciences

## Introduction

Meniere disease (MD) is a debilitating disorder of the inner ear, characterized by vertigo episodes, hearing loss, and tinnitus.^1^ Although predominantly diagnosed as sporadic, up to 10% of cases exhibit familial aggregation, indicating a significant genetic contribution.^2^ Investigations into the genetic structure of familial MD (FMD) have successfully identified rare variants in several genes, including *OTOG*, *MYO7A*, and *TECTA*, which mediate dominant and recessive inheritance through by one or more heterozygous variants in the same gene.^3^ These studies have established that FMD is genetically heterogeneous.^4^ Furthermore, comparative analyses suggest that the genetic landscape of familial and sporadic MD (SMD) is distinct, with familial cases showing a greater burden of rare and functionally significant variants in auditory and vestibular genes^5^^,^^6^ Despite these advances, a large fraction of FMD cases remains without a definitive molecular diagnosis, highlighting the complexity of its genetic structure^7^

The increasing volume and complexity of genomic data generated necessitates advanced analytical approaches to integrate clinical phenotypes and prioritizes causative variants.^8^^,^^9^ The application of machine learning (ML) models in biomedical research has increased our understanding of the genetic structure of rare genetic disorders by enabling scalable analysis of high-dimensional omics data. ML models have demonstrated substantial advances in variant prioritization, disease classification, and identification of multiallelic inheritance patterns.^10^ Systematic reviews have emphasized the growing relevance of ML in diagnosing rare diseases, especially those with elusive or polygenic etiologies.^11^ Computational classifiers include multiple features to prioritize several types of rare variants of unknown significance in coding regions.^12^ Moreover, a disease-specific prediction tool for variant prioritization was designed for genetic diagnosis of hearing loss using a hearing loss panel of 157 genes.^13^

However, despite the availability of such genetic data, no prior work has attempted to stratify MD patients using supervised ML models based on comprehensive variant annotations using rare variants and exome sequencing datasets. To our knowledge, this is the first study to leverage supervised ML techniques to classify MD patients as familial or sporadic based solely on exome sequencing data, with the aim of uncovering hidden familial predisposition in phenotypically sporadic cases. Our approach builds upon this gap by integrating gene burden-based scores, protein-protein interaction metrics, variant impact scores (such as combined annotation-dependent depletion [CADD] PHRED), and phenotype annotations into an interpretable feature set used to train classification models (Table 1). By leveraging coding sequence data, we aim to uncover “hidden inheritance” patterns in individuals diagnosed with the sporadic form of the disease, an approach that could be transformative for genetic counseling and patient management in rare diseases.

**Table 1:** Feature matrix computed to train the Meniere disease classifier

Gene and variant features	Disease-specific features
**Gene variant burden score (GVBS)**	**Meniere phenotype score (MPS)**
Quantitative score represents the overall genetic burden of rare variants within a specific gene; it is calculated using a custom formula that considers the number of rare variants (nVarks), their allele frequencies (AFs), and the length of the gene	This is a binary flag (1 or 0) that indicates if a gene has a known association with at least one of the target phenotypes (vertigo, hearing loss, and tinnitus); the annotation is derived from querying the MouseMine database
**CADD PHRED score**	**Associated phenotype number (APN)**
This score estimates the pathogenic impact of a specific variant; the score for each variant was annotated using the combined annotation dependent depletion (CADD) tool	This is a quantitative count (0, 1, 2) of how many of the target phenotypes are associated with a single gene, based on data from the MouseMine database
**Biallelic variant interaction score (BVIS)**	
This score models the potential for biallelic inheritance by calculating the product of allele frequencies of two different co-occurring variants (AF_1_ × AF_2_); it is designed to capture the combined effect of two separate variants that, when inherited together, may contribute to the disease phenotype	
**Protein-protein interaction (PPI)**	
The confidence score of physical interactions between the protein products of two different genes; it was derived from the STRING database and is used to model potential digenic relationships	

## Results

### Rare variant distribution in familial and SMD datasets

Figure 1A shows the relative frequency of rare variants in FMD and SMD cases according to their predicted functional impact. Missense and synonymous variants were the most common types of single nucleotide variants (SNVs) identified in both familial and sporadic datasets (Figure 1B), and comparisons are presented by clinical label. Since family history may be under-ascertained, distributional contrasts by clinical label could be attenuated by label noise. However, the overall genetic structure showed a significantly different distribution according to variant types. Specifically, missense variants with moderate or modifier impact were more prevalent in the clinically defined sporadic group, suggesting the hypothesis of hidden recessive inheritance. Furthermore, loss-of-function (LoF) variants were also enriched among sporadic cases, suggesting the presence of undetected dominant or semidominant effects.

**Figure 1:**
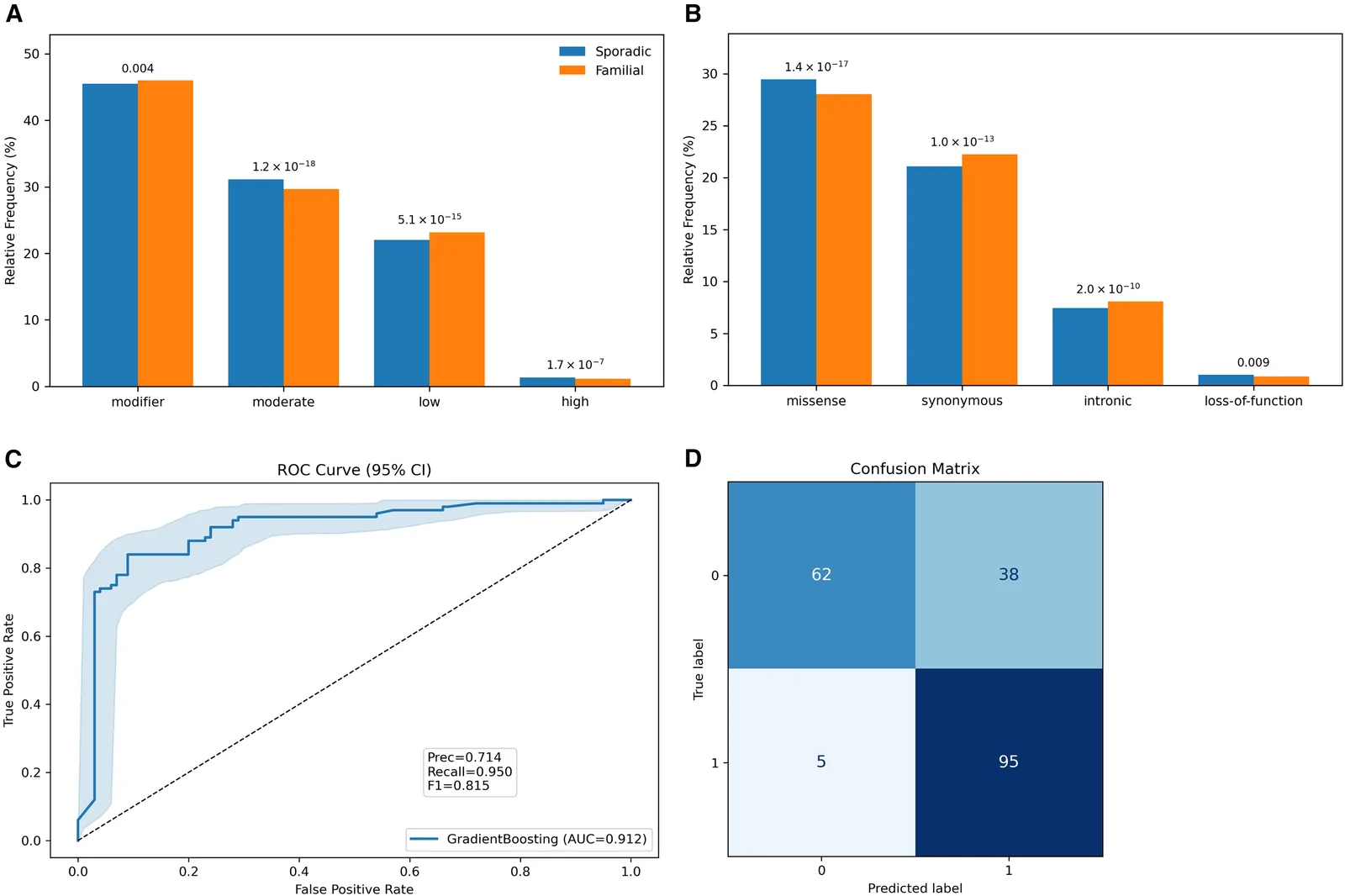
Distribution of rare variants in familial and sporadic MD and performance of the classification model (A) Relative frequency of rare variants in familial and sporadic MD cases according to their predicted functional impact: high, moderate, low, or modifier. (B) Relative frequency of rare variants according to their annotated functional consequence: missense, synonymous, intronic, or loss-of-function. Differences in variant frequencies between familial and sporadic cases were assessed using the chi-squared test with Yates’ correction, and FDR-adjusted *p* values are shown above the corresponding bars. (C) Receiver operating characteristic (ROC) curve, with 95% confidence interval, for the gradient boosting model developed using rare-variant features to distinguish familial from sporadic MD cases. The model achieved an area under the curve (AUC) of 0.912; precision, recall, and F1 score at the selected classification threshold were 0.714, 0.950, and 0.815, respectively. The dashed diagonal line represents performance expected by chance. (D) Confusion matrix showing the number of observations assigned to each true and predicted class at the selected classification threshold (0 familial, 1 sporadic). Thirty-eight percent of clinically defined sporadic cases were classified as familial MD.

### Familial/sporadic classification using exome sequencing datasets

The performance of supervised ML models for classifying patients with MD as either familial or sporadic based on a curated genomic feature matrix is presented in Figure S1. Among the evaluated models, gradient boosting (GB) demonstrated the highest discriminative ability on the held-out test set, achieving a receiver operating characteristic-area under the curve (ROC-AUC) of 0.843 (95% confidence interval [CI]: 0.706–0.948), an average precision (AP) of 0.941 (95% CI: 0.867–0.990), and an accuracy of 81.5% (95% CI: 72.3–90.8%). The random forest (RF) model showed comparable performance, with an ROC-AUC of 0.843 (95% CI: 0.699–0.951), AP of 0.939 (95% CI: 0.858–0.990), and an accuracy of 80.0% (95% CI: 69.2%–89.2%). In contrast, the logistic regression (LR) model, while serving as a useful linear baseline, yielded comparatively lower performance metrics, including an ROC-AUC of 0.606 (95% CI: 0.433–0.758), AP of 0.853 (95% CI: 0.739–0.947), and accuracy of 63.1% (95% CI: 50.8–73.9%). Although both tree-based models (GB and RF) demonstrated superior training performance, their wider confidence intervals and reduced generalization on the test set indicate potential overfitting. Conversely, the LR model exhibited more stable variance but at the cost of limited discriminative power. Taken together, considering its favorable balance between discrimination, calibration, and generalization, the GB classifier was selected as the optimal model for downstream analysis (Figure 1C). Moreover, the GB classifier was able to correctly classify 95% of familial cases, but also re-classified 38% of clinically defined sporadic cases as FMD (Figure 1D).

### Validation of the GB-based model

To evaluate generalizability, the GB classifier was tested on an independent cohort of 100 previously unseen patient files (50 familial and 50 SMD), aggregated using the same variant- and gene-level feature pipeline. The model achieved an external ROC-AUC of 0.880, AP of 0.886, accuracy of 76%, and a Brier score of 0.138, closely matching internal test performance (ROC-AUC = 0.843). The classification report showed high recall for sporadic patients (88%), confirming the model’s ability to capture latent familial predisposition among clinically sporadic cases. These results reinforce the robustness and translational potential of the GB model for distinguishing hidden inheritance patterns in MD. Since we did not perform trio-based phasing or segregation; thus, the familial-like designation reflects model-based prioritization rather than confirmed recessive inheritance.

### Familial predisposition in SMD individuals

To simulate a clinical scenario, we applied the final GB model to a subset of 100 clinically diagnosed SMD patients. Of these, 28 individuals (28%) were reclassified as familial cases, indicating a hidden genetic signature consistent with familial inheritance. This demonstrates the model ability to uncover rare genetic variation and a latent familial predisposition even when family history is absent. When applied to the entire sporadic cohort from the original dataset (*n* = 336), the model identified 95 patients as familial-like, corresponding to 28.3% of the cohort. This proportion was calculated by applying the optimized decision threshold (Youden’s J = 0.348) derived from the validation set and counting the number of sporadic patients with predicted probabilities exceeding this threshold. Reporting the result both as an absolute count (95 of 336) and a proportion (28.3%) provides clarity and reproducibility.

We also tested the GB model with 6 FMD individuals with known mutations in the genes *OTOG*, *MYO7A*, *ADGRV1*, *GJD3*, and *TECTA* and all were correctly classified as familial cases. A complete list of genes and variants found in the Spanish families is shown in Table 2.

**Table 2:** Genes and variants identified in the 93 Spanish families with documented history of Meniere disease in at least 2 individuals

Genes	Variants	Number of FMD cases^a^	ACMG	AF gnomAD		Family code	Reference
				NFE	Global		
*TIMCC*	chr2:70300842 G>A	3	LP	–	–	F1	Requena et al.^14^
*DTNA*	chr18:34882130 G>T		VUS	3.39E-06	1.24E-05		
*PRKCB*	chr16:23988577 G>T	3	P	–	6.20E-07	F2	Martin-Sierra et al.^15^
*SEMA3D*	chr7:85012812 G>T	3	P	–	–	F3	Martin-Sierra et al.^16^
*DPT*	chr1:168696611 C>T	3	P	–	–	F4	
*OTOG*	chr11:17557227 G>A	5	VUS	0.003	0.007	F5, F6, F7, F8	Roman-Naranjo et al.^17^
	chr11:17611374 C>T	5	VUS	0.001	0.002		
	chr11:17642200 G>A	5	VUS	0.002	0.002	F9, F10, F11	
	chr11:17645592 G>C	6	LB	0.021	0.018	F12, F13, F14	
	chr11:17553211 G>A	3	LP	0.001	0.001	F9, F11	
	chr11:17599671 C>T	2	VUS	0.006	0.005	F15, F16	
	chr11:17573200 C>A	2	VUS	–	–	F17	
	chr11:17606001 G>A	1	VUS	0.004	0.003	F11	
	chr11:17609906 C>T	1	LB	0.012	0.011	F18	
	chr11:17635125 G>A	1	VUS	0.005	0.004	F19	
	chr11:17611118 C>T	1	VUS	5.23E-06	3.87E-06	F20	Parra-Perez et al.^18^
	chr11:17641065 G>A	1	VUS	1.22E-05	1.10E-05	F21	
	chr11:17612217 G>A	1	VUS	0.001	0.001	F22	
	chr11:17610645 T>C	1	LB	0.004	0.003	F23	
	chr11:17640936 C>T	1	B	1.76E-04	0.001	F24	
	chr11:17638480 C>A	1	B	9.50E-05	0.001	F25	
*MYO7A*	chr11:77130637 G>A	3	LP	–	4.34E-06	F5	Roman-Naranjo et al.^19^
	chr11:77174877 G>A	1	VUS	7.63E-05	1.43E-04	F26	
	chr11:77199601 G>A	1	LP	–	–	F27	
	chr11:77214688 G>A	1	B	2.32E-04	0.004	F28	
*MYO7A*	chr11:77179874 G>A	1	VUS	1.92E-05	3.12E-05	F6	
*ADGRV1*	chr5:90694338 C>T		VUS	0.003	0.004		
*MYO7A*	chr11:77180404 C>T	2	VUS	0.001	0.001	F29	
*ADGRV1*	chr5:90840606 G>A		VUS	3.82E-05	6.46E-05		
*MYO7A*	chr11:77159450 G>A	2	VUS	0.003	0.002	F30	
*CDH23*	chr10:71732116 A>G		VUS	0.006	0.005		
*MYO7A*	chr11:77214674 G>A	2	VUS	1.12E-05	1.52E-05	F31	
*PCDH15*	chr10:53822490 G>T		VUS	1.23E-05	1.28E-05		
*MYO7A*	chr11:77211830 G>A	1	VUS	4.06E-04	4.31E-04	F32	
*USH1C*	chr11:17509546 G>C		VUS	0.001	0.001		
*TECTA*	chr11:121158016 T>C	5	VUS	6.53E-05	6.88E-05	F33, F34	Roman-Naranjo et al.^20^
	chr11:121152980 G>C	2	VUS	5.93E-06	1.24E-05	F35	
	chr11:121157957 AC>A	1	LP	–	–	F36	
	chr11:121165368 C>T	1	VUS	–	6.28E-07	F37	
	chr11:121189865 GC>C	1	LP	–	–	F38	
*GJD3*	chr17:40356228 C>T	5	VUS	0.024	0.020	F39, F40, F41	Escalera-Balsera et al.^21^
	chr17:40363058 C>G		LB	0.024	0.021		
	chr17:40363293 G>A		LB	0.024	0.021		
	chr17:40363294 C>G		LB	0.024	0.021		
	chr17:40363579 G>T		LB	0.024	0.020		
*TRIOBP*	chr22:37769343 C>T	2	VUS	3.22E-05	2.86E-05	F42	Cruz-Granados et al.^22^
*KIF1B*^b^	chr1:10374335 C>T	1	VUS	3.73E-05	3.04E-05	F43	Cruz-Granados et al.^23^

Fifty familial cases remain unresolved.

a Number of familial MD cases with confirmed variant across one or more families.

b Identification in unaffected family members could not be performed due to lacking exome data.

Furthermore, when the model was applied to exome sequencing data from individuals with chronic tinnitus but without a diagnosis of MD, used as pseudo-controls it failed to correctly classify these files (Figures S2A and S2B). When random labels were assigned to non-MD files, the resulting distribution was centered at chance level (mean AUC ∼0.50), demonstrating that the classifier cannot discriminate among files when the true signal associated with MD genomic structure is not present; mean predicted probability = 0.69 ± 0.07, with all samples incorrectly assigned as Meniere-like) (Figure S3). This result supports the specificity of the engineered genomic features for the genetic structure of MD, according to the rare variant distribution, rather than reflecting general auditory phenotypes.

### Feature ablation analysis in the GB model

Ablation analysis shows that removing the biallelic variant interaction score (BVIS) (baseline 6 features → 5 features) produced the largest performance degradation across nearly all metrics (Table S1), with substantial declines in discrimination and classification quality (ΔTEST_AUC = −0.387, ΔTEST_AP = −0.196, ΔTEST_REC1 = −0.157, ΔTEST_PREC1 = −0.076, ΔTEST_F1_1 = −0.117, ΔTEST_BAL_ACC = −0.186, ΔTEST_MCC [Matthews correlation coefficient] = −0.397) and a marked worsening in calibration (ΔTEST_BRIER = +0.106), indicating BVIS is a key driver of model performance and emphasizing that the familial-like label is driven by a biologically motivated signal. Removing the CADD PHRED score yielded a smaller but consistent drop in performance (ΔTEST_AUC = −0.039, ΔTEST_AP = −0.016, ΔTEST_BAL_ACC = −0.062, ΔTEST_MCC = −0.094) and worse calibration (ΔTEST_BRIER = +0.015). Dropping the gene variant burden score (GVBS) resulted in modest changes overall (ΔTEST_AUC = −0.027, ΔTEST_AP = −0.032, ΔTEST_BRIER = +0.010) with slight improvements in recall and F1 (ΔTEST_REC1 = +0.059, ΔTEST_F1_1 = +0.014) but reduced precision (ΔTEST_PREC1 = −0.023). Removing protein-protein interaction score (PPIS) had minimal impact across most measures (ΔTEST_AUC = −0.010, ΔTEST_AP = −0.004, ΔTEST_BRIER = +0.003) and shows small improvements in balanced accuracy and MCC (ΔTEST_BAL_ACC = +0.026, ΔTEST_MCC = +0.028), suggesting PPIS is comparatively less influential in this dataset.

In contrast, removing the phenotype-derived block features (Meniere phenotype score [MPS] and associated phenotype number [APN]) (baseline 6 features → 4 features)—increased overall discrimination (ΔTEST_AUC = +0.029) and substantially improved threshold performance in terms of balanced accuracy and MCC (ΔTEST_BAL_ACC = +0.155, ΔTEST_MCC = +0.181) while improving calibration (ΔTEST_BRIER = −0.014); however, this was associated with a recall–precision trade-off (ΔTEST_REC1 = −0.118 vs ΔTEST_PREC1 = +0.108), indicating that phenotype removal shifts the operating behavior toward fewer false positives at the expense of more false negatives under the selected threshold.

### Bi-allelic inheritance in MD

Since the BVIS was found to be the best predictor to the GB model, we retrieved and ranked genes according to BVIS for FMD and SMD to determine which genes contribute to the biallelic inheritance. Although FMD and SMD showed a divergent genetic structure for this feature, 359 genes were shared between both subsets. Normalized delta *p* values for top-ranked genes for FMD and SMD highlight genes exclusively contributing to either FMD (*COL4A4*, *DOCK6*, and *SLC29A3*) or SMD (*PPL*, *MICAL1*, and *DCHS1*) (Figure S2; Tables S2, S3, and S4).

### Pathway analysis in FMD and SMD

When pathway analysis was performed in the top genes according to the biallelic scores and CADD scores (Tables S5 and S6), we found that there were differences between FMD and SMD (Figures 2 and 3). In FMD, Kyoto Encyclopedia of Genes and Genomes (KEGG) and Reactome unique pathway enrichment was ion transport and extracellular matrix degradation, while Gene Ontology (GO) terms showed polarized cell growth and ATPase/transporter activities. SMD showed unique enrichment of related immune KEGG pathways such as human papillomavirus infection and integrin signaling, Reactome pathways involving immune receptor diversification, and GO terms related to cilium organization and sensory perception. Shared enrichment across FMD and SMD included cytoskeleton organization and extracellular matrix remodeling pathways identified by KEGG, Reactome, and GO analyses.

**Figure 2:**
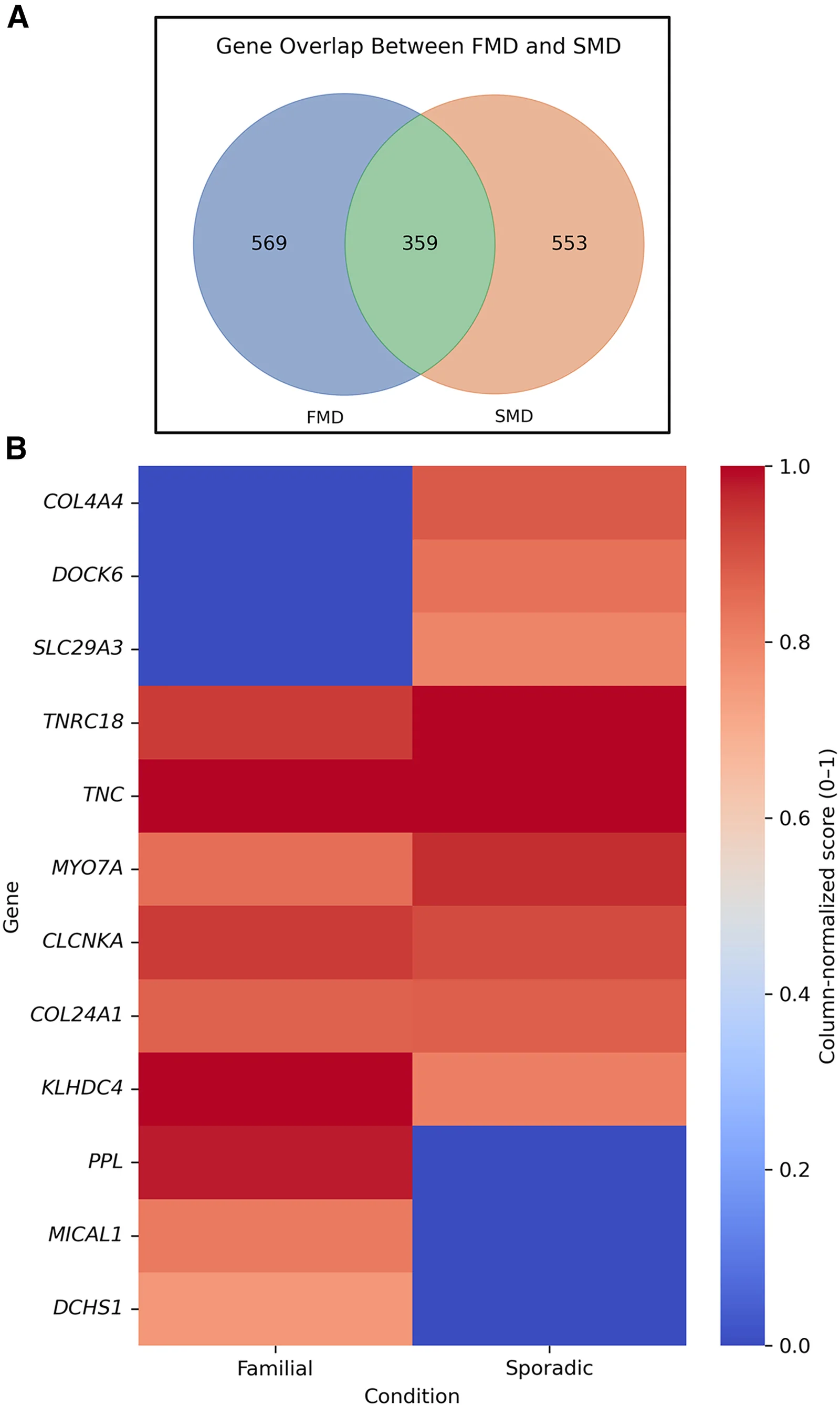
Genetic structure according to rare variant distribution in familial and sporadic MD (A) Venn diagram showing the number of genes contributing to biallelic inheritance in MD. (B) Heatmap showing genes that mostly contributed to BVIS in familial or sporadic MD. Biallelic variant interaction scores were normalized for each gene to compare FMD and SMD.

**Figure 3:**
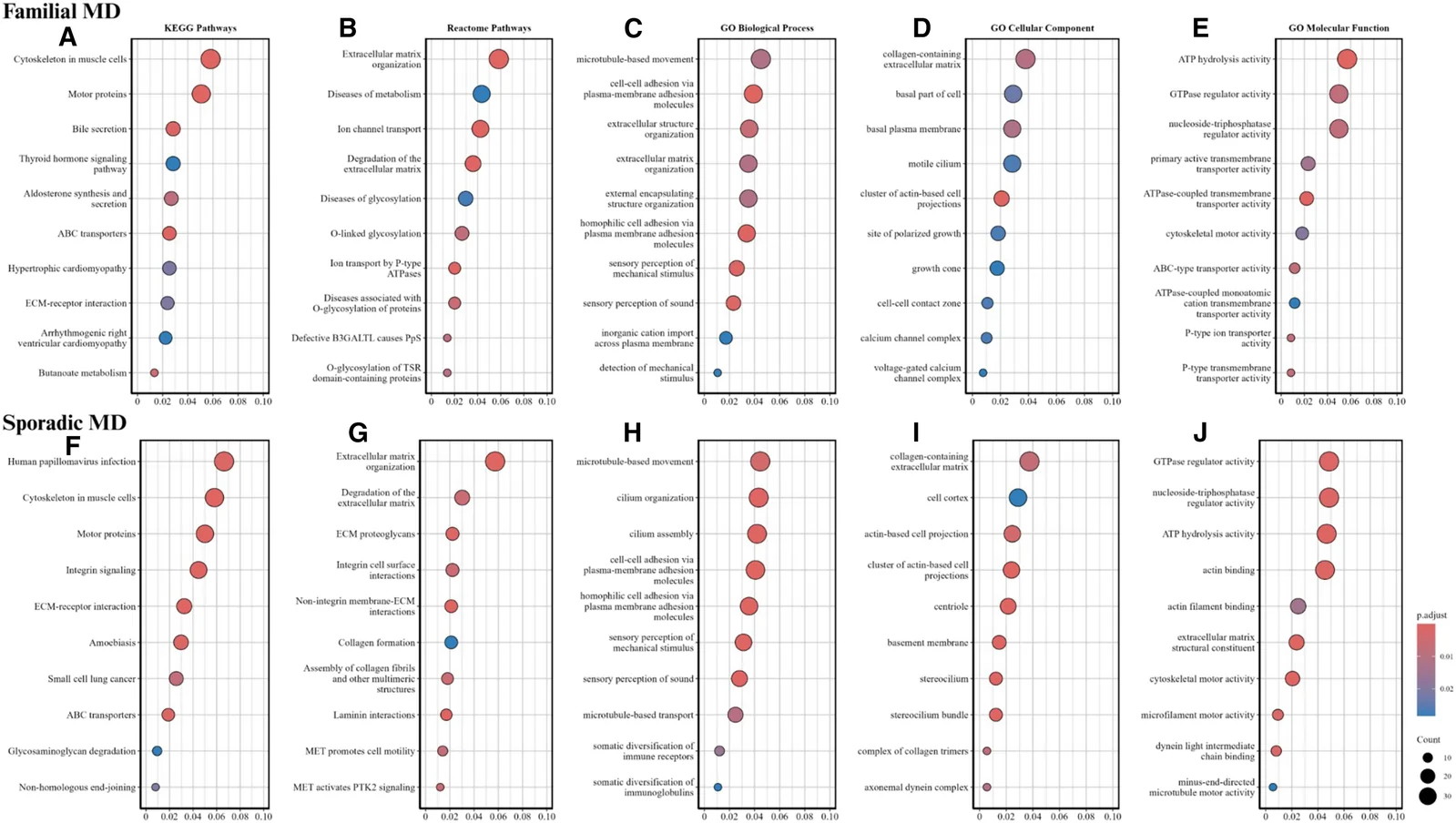
Top 10 enriched pathways (FDR < 0.05) The top row represents pathways enriched in familial Meniere disease (MD) and the bottom row represents pathways enriched in sporadic MD. Images show results from different analyses: (A–F) KEGG pathways; (B–G) Reactome pathways; (C–H) Gene Ontology (GO) biological processes; (D–I) GO cellular component; and (E–J) GO molecular function.

## Discussion

This study provides the MD classifier, a novel ML approach using rare variation and exome datasets, able to uncover a burden of biallelic heterozygous variants in the same genes suggesting hidden recessive inheritance in the clinically defined sporadic group (Figure 4). The application of supervised ML models to analyze the interaction of missense and synonymous variants in large-scale genomic MD datasets has not been previously explored. Previous studies have shown that both FMD and SMD have a burden of missense and LoF variants in genes linked audio-vestibular phenotypes;^5^^,^^6^ nevertheless, the burden of missense and LoF variants is higher in FMD individuals, suggesting a divergent genetic structure according to the rare variant distribution.^6^ By using the different distribution of rare variant in FMD and SMD, we designed a set of gene and variant features (GVBS, BVIS, and PPIS), phenotype features (MPS, APN) and used CADD (as measure of variant functional impact) to build and train several ML models that could classify individual variant call format (VCF) files as FMD or SMD. Among these models, the GB exhibited the best performance (AUC = 0.843) and it was able to re-classify 28% of SMD as FMD.

**Figure 4:**
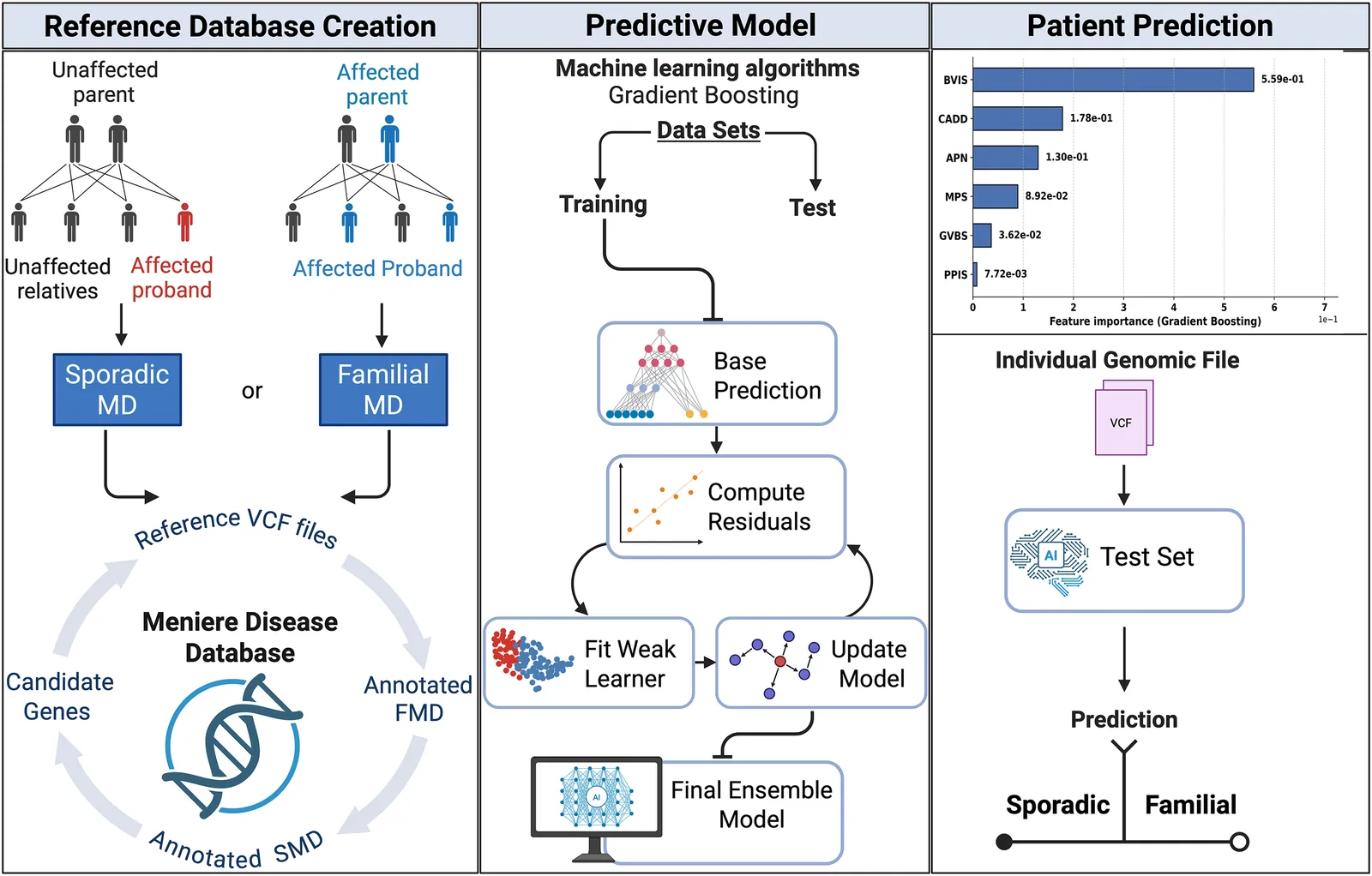
Flowchart with the architecture of the Meniere disease classifier using rare variants in the coding regions in familial or sporadic cases A dataset of 429 exome sequencing vcf files were used to generate the reference dataset. The ML predictive model was designed using the following features BVIS, biallelic variant interaction score; CADD, combined annotation-dependent depletion; APN, associated phenotype number; MPS, Meniere phenotype score; GVBS, gene variant burden score; PPIS, protein-protein interaction score.

These results may have significant impact on the diagnosis and management of MD, raising the proportion of genetic cases up to 28% within individuals without a familial history of MD. FMD exhibits a large genetic heterogeneity,^21^^,^^24^ and it has been reported in 6% of East Asians^25^ and 9% of Southern Europeans. Genomic research has reported ∼20 candidate genes in FMD with monoallelic and biallelic inheritance.^24^ American and European clinical practice guidelines for MD recognize FMD, but they do not include any recommendation on genetic testing.^26^ Given that >25% of SMD share the same genetic structure with FMD, and in the light of gene therapy development for hearing loss in humans,^27^ up to 38% of MD patients could potentially benefit from a genetic diagnosis and hopefully gene therapy in the future.

Overall, the ablation analysis indicates that BVIS provides the strongest and most consistent contribution to GB performance, CADD PHRED score and GVBS contribute smaller but non-negligible signals, PPIS has the smallest marginal effect in this cohort, and the phenotype block (MPS+APN) appears partially redundant and can alter the recall-precision balance. Given the relatively wide confidence intervals in several metrics, especially for MCC, modest differences should be interpreted cautiously and framed as dataset-dependent.

BVIS was the most important feature of the ML model. BVIS computes all pairwise combination of rare variants in the same gene (AF < 0.01), including missense and synonymous variants. Recessive inheritance associated with compound heterozygous variants is frequently observed for missense variants in hearing loss genes,^28^ and it has been reported for *OTOG* in FMD.^18^ We hypothesize that missense and synonymous variants in the same gene may have an additive effect with functional consequences, and this feature was exploited by the GB model to classify individuals as FMD or SMD. According to this feature, biallelic inheritance could explain a significant proportion of the inheritance observed in FMD.

Data analysis also revealed a significant difference in CADD scores between familial and sporadic cases, making it a crucial predictor for the classification model.^29^ This feature provides an additional layer of biological relevance, capturing the potential pathogenic impact of each variant and improving the discriminatory power of ML models.^30^

We used the BVIS feature to identify genes associated with either FMD or SMD. Although 359 genes overlapped between both the datasets, their contribution to FMD or SMD was different. To assess the importance of BVIS in FMD, we compared the 200 top-ranked genes according to the BVIS Dp value with the FMD genes with the burden of rare variation previously reported that predicted high or moderate impact on the protein.^6^ Among the 200 top-ranked BVIS-prioritized genes, 60 were identified as FMD genes, indicating that approximately 30% of FMD genes may be influenced by biallelic inheritance. Moreover, when we compared them with the SMD genes showing a burden of rare variation, 56 SMD genes were also found in the 200-ranked BVIS-prioritized genes with an overlap of 22 genes between FMD and SMD. Collectively, this gene overlap suggests that biallelic inheritance could partially explain hidden inheritance in MD.

The ablation analysis also showed that removing the phenotype-derived block features (MPS +APN) improves several overall test metrics, even though one metric worsens. Compared with the baseline, dropping MPS+APN increases TEST_AUC by +0.029, improves TEST_BAL_ACC by +0.155, improves TEST_MCC by +0.181, and improves calibration (TEST_BRIER decreases by −0.014), indicating better overall separation and better-calibrated probabilities under the selected thresholds. However, this improvement is not uniform; it comes with a recall-precision trade-off, where TEST_REC1 decreases by −0.118 while TEST_PREC1 increases by +0.108. The most defensible explanation is that MPS/APN add noisy or partially redundant signal in this dataset and removing them reduces variance/overfitting and shifts the model toward a more conservative decision behavior (fewer positives predicted), which improves precision, balanced accuracy, MCC, and calibration but reduces sensitivity (recall). In other words, “improvement after removal” does not mean MPS/APN are biologically irrelevant; rather, under the current data and thresholding procedure, they appear to destabilize probability estimates and/or push the classifier toward more false positives, and removing them yields a cleaner, better-calibrated model that prioritizes precision over recall. Moreover, the lower performance of the classifier without phenotype features also suggests that phenotype annotation is incomplete and most of the genes associated with auditory of vestibular phenotype,^31^ including FMD genes have not been discovered.^24^

The top 1,000 genes were ranked for both BVIS and CADD scores and were considered for the FMD and SMD pathway enrichment analyses. In a cohort 511 MD patients, Fisch et al.^4^ identified 1,098 highly recurrent genes (≥ 4 ultra-rare variants), which were significantly enriched for GO terms that belong to the cell junction function, ATP binding, and actin binding. Network propagation (network *Z* > 3 and recurrence ≥4; *N* = 481 genes) confirmed enrichment for the ATP binding term, myosin complex term, and cytoskeleton organization term. In a study by Parra-Perez et al.,^6^ genes showing variants in ≥3% of the population were enriched for cell-cell junction and microtubule terms in the case of SMD patients and cell projection, myosin complex, and cytoskeleton organization terms were enriched for FMD patients.

In contrast, our results using BVIS/CADD showed a different pattern of enrichment. In SMD, GO term pathways were associated with cilium organization and sensory perception, and KEGG/Reactome showed immune receptor diversification. In FMD, GO pathways were associated with polarized cell growth and ATPase/transporter activity, with KEGG/Reactome showing ion transport.

While 80% of rare diseases are estimated to be caused by genetic factors, the etiology of a significant number of these diseases remains unknown or poorly characterized.^32^ Here, we demonstrate that despite the complex genetic structure of the condition, several ML models can reasonably classify MD patients into familial or SMD, exclusively using rare variation. Our findings not only confirm the feasibility of this approach but also suggest its potential utility in other rare diseases with genetic heterogeneity.

The results generated by the MD classifier support the hypothesis of “hidden recessive inheritance in the clinically defined sporadic group,” where individuals diagnosed with SMD may carry a burden of rare variants that predisposes them to the disease, potentially by multiallelic interactions.

The identification of biallelic heterozygous variants in the same genes has profound implications for genetic counseling and patient management in MD also. It suggests that the genetic architecture of MD can be multiallelic, involving one or multiple genes. Remarkably, the importance of BVIS in the classifier suggests a combined effect of missense and synonymous variants in the same genes and supports the inclusion of synonymous variants on segregation analysis together with missense variants of unknown significance. The features used for this MD classifier, could be adapted for the study of other genetically heterogeneous rare diseases with familial and sporadic cases.

These features used by the ML classifier support a distinct genetic architecture for FMD and SMD but also confirm an overlap with ∼300 genes with a different density of rare variants. The features and model architecture show the importance of biallelic heterozygous inheritance in MD with European ancestry. This ML classifier could be applied to other rare disorders with familial and sporadic cases to identify hidden inheritance patterns.

### Limitations of the study

Several limitations should be considered when interpreting our findings. First, we could not perform trio-based phasing or segregation; thus, the familial-like designation reflects model-based prioritization rather than confirmed recessive inheritance; second, MD exome datasets available were limited to European population and we could not assess if our model will also keep the same accuracy with other populations. Future work will focus on validating this classifier in the other independent MD cohorts. Third, while we included multiple datasets, a large sample size through collaborative multi-site consortia would enable more precise detection and evaluation of other neurological conditions with familial and sporadic cases (i.e., Parkinson’s disease, Alzheimer’s disease) to reveal hidden inheritance associated to rare multiallelic inheritance. Finally, the MD classifier cannot identify pathogenic variants for a genetic diagnosis. Its potential applicability would be limited to classify individuals as familial or sporadic.

## Resource availability

### Lead contact

Further information and requests for resources should be directed to and will be fulfilled by the lead contact, Jose A. Lopez-Escamez (jose.lopezescamez@sydney.edu.au).

### Materials availability

This study did not generate any new unique reagents. Information about data collection and data analysis is available from the lead contact upon request.

### Data and code availability

• All associated data with this paper are listed in the key resource table. No new data have been generated in this study. This paper analyses existing publicly available data on the European Genome-Phenome Archive (EGA); all dataset accession numbers are listed and referenced in key resources table.
• Data and code used during the development of this study are available on GitHub at https://github.com/MDNLAtlas/MD-Classifier.
• Requests for further information may be directed to the lead contact.

## Acknowledgments

We thank all clinicians in the Meniere Disease Consortium for the effort to identify and recruit the Spanish Meniere disease cohort and the past members of Lopez-Escamez lab for their methodological support and regular insightful discussions. We sincerely thank Mai T. Pham for her careful review and suggestions to improve the manuscript.

This research has been funded by the K7013-B3414G grant from 10.13039/501100001774The University of Sydney. Pablo Cruz-Granados is funded by The Garnett Passe and Rodney Williams research scholarship (SC5822).

## Author contributions

S.A.M., data curation, formal analysis, software, writing – original draft, and writing – review and editing; M.H.B.K., data curation, formal analysis, software, visualization, writing – original draft, and writing – review and editing; P.C.-G., formal analysis, visualization, writing – original draft, and writing – review and editing; K.B.-L., software, writing – original draft, and writing – review and editing; A.B., writing – review and editing; J.A.L.-E., conceptualization, funding acquisition, supervision, writing – original draft, and writing – review and editing.

## Declaration of interests

The authors declare no competing interests.

## STAR★Methods

### Key resources table

**Table:** 

REAGENT or RESOURCE	SOURCE	IDENTIFIER
**Deposited data**		

Familial Meniere disease cohort raw data	European Genome-Phenome Archive (EGA)	EGAD50000001682
Sporadic Meniere disease cohort raw data	Mendeley data	https://doi.org/10.17632/8dgfvbyyrk.1
Homo sapiens reference genome (GRCh38)	Genome Reference Consortium	https://www.ncbi.nlm.nih.gov/grc/human

**Software and algorithms**		

MD Classifier	This paper; GitHub	https://github.com/MDNLAtlas/MD-Classifier
Variant Effect predictor	McLaren et al.^24^	https://useast.ensembl.org/vep?
STRING	Szklarczyk et al.^33^	https://string-db.org/
MouseMine	Motenko et al.^27^	https://www.mousemine.org/mousemine/begin.do
ClusterProfiler v.4.14.6	Wu et al.^34^	https://bioconductor.org/packages/release/bioc/html/clusterProfiler.html
ReactomePA v.1.50.0	Yu et al.^35^ Milacic et al.^36^	https://www.bioconductor.org/packages/release/bioc/html/ReactomePA.html
Imblearn v1.14.2	Lemaître et al.^37^https://jmlr.org/papers/v18/16-365.html	https://imbalanced-learn.org/stable/references/generated/imblearn.pipeline.Pipeline.html

### Experimental model and study participant details

#### Human datasets

The experimental protocol of this study was approved by the Institutional Review Board (PE-0356-2018). Each participant signed a written informed consent before donating samples. The study was carried out according to the principles of the Declaration of Helsinki for investigation with human subjects.

written informed consent form. This study utilized a publicly available whole exome sequencing (WES) dataset comprising 429 unrelated patients, consisting of 93 FMD cases (EGAD50000001682) and 336 sporadic cases (https://doi.org/10.17632/8dgfvbyyrk.1)^6^ All included individuals were over 18 years old.^6^ The diagnosis of MD was previously established according to the diagnostic criteria outlined by the International Classification Committee for Vestibular Disorders of the Barany Society in 2015.^1^

A set of 101 WGS files from the UNITI cohort, consisting of individuals with tinnitus, but without diagnosis of MD,^38^ was retrieved to test the performance of the Gradient Boosting model.^13^ This non-MD cohort was used to confirm that the performance of the model to classify MD patients was not observed in individuals with other hearing or brain disorders.

### Method details

#### Feature engineering

Figure 4 show the architecture of the datasets and the workflow of the classifier. To prepare the data for the supervised ML, several variables were engineered to capture distinct variant-related and phenotype-related features from the genetic dataset.

1. Gene Variant Burden Score (GVBS)

To reduce dimensionality while preserving biological relevance, rare variants were collapsed at the gene level to compute a Gene Variant Burden Score (GVBS) for each individual. This GVBS integrates variant count, gene length normalization, allele frequency burden, and disease prevalence into a unified metric. For each gene k, the VBS was calculated as:

$$GVBS_{k}=n_{k}\times \left(L_{k}\right)/\left(L_{m}ed\right)\times \min\left({\left(\left(\prod^{A}F_{i}\right)/\mathrm{Pr}\;e\;valence\right)}^{\left(-1\right)},1\right)$$

Where n_k_ is the number of rare variants in gene k; L_k_ is the length of gene k; L_m_ed is the median gene length across all genes; AFᵢ is the allele frequency of the i-th variant; and Prevalence is the estimated disease prevalence.^7^

2. Variant Functional Impact

The CADD PHRED score was used to estimate the predicted functional consequence of each variant at the protein sequence. Scores were grouped into Maximum Impact for Loss-Of-Function (LoF) variants (e.g., frameshift, nonsense, splice-site), Moderate Impact (missense), Low Impact (e.g., UTRs), and No Significant Impact (e.g., intronic, synonymous). Missense variant leading to splice-site were classified as LoF.

3. Modeling biallelic Inheritance
  To account for biallelic inheritance, the following features were computed
  a. Biallelic Variant Inheritance Score (BVIS): For each gene in an individual, BVIS was computed as the count of all pairwise combinations of rare variants (AF < 0.01) for which the allele frequency product (AF_1_ × AF_2_) was less than or equal to 0.001, with the assumption of variant independent inheritance and according to the estimated MD prevalence. This feature models the joint probability of biallelic inheritance and captures the potential burden of digenic or compound heterozygous variant effects.
  b. Protein–Protein Interaction Score (PPIS): For each gene, the maximum PPIS from the STRING database (v12.0) was used to capture gene-level connectivity, reflecting potential digenic or modifier roles.^33^
4. Phenotype-Informed Features
  To enrich the dataset with phenotype relevance, variants were mapped to genes associated with inner ear or vestibular phenotypes (e.g., hearing loss, vertigo, tinnitus) using curated sources including MouseMine.^39^ Two features were derived:
  a. Meniere Phenotype Score (MPS): A binary feature (1 or 0) indicating if the gene was associated with a relevant phenotype.
  b. Associated Phenotype Number (APN): The total number of phenotype-associated variants in a given gene for each individual.

#### Final dataset structure

The final dataset used for ML was structured as a feature matrix where each row represents a patient and each column represents a feature. These features were engineered to capture distinct biological and genetic information necessary for the classification model. The key features are shown in Table 1.

#### Machine learning approach for MD classification

A leakage-safe machine learning pipeline was implemented with a stratified train/validation/test split (70/15/15). Class imbalance was addressed by applying Synthetic Minority Oversampling Technique (SMOTE) inside each cross-validation (CV) fold using an Imblearn v1.14.2^37^ pipeline, ensuring no information leakage between training and evaluation.

Five supervised classifiers—Gradient Boosting (GB), Random Forest (RF), Logistic Regression (LR), Support Vector Machine (SVM-RBF), and k-Nearest Neighbors (KNN)—were trained and compared. For LR, SVM, and KNN, feature scaling was applied using StandardScaler. Model hyperparameters were tuned using GridSearchCV with Stratified 5--Fold CV, optimizing ROC-AUC as the primary metric.

A three-phase evaluation strategy was used: (i) hyperparameter optimization on the training set with internal cross-validation; (ii) threshold selection on the validation set using Youden’s J statistic; and (iii) final unbiased evaluation on the held-out test set. For each model, performance was quantified using accuracy, precision, recall, ROC-AUC, and Brier score. Bootstrapping (*n*=1000) was applied to compute 95% confidence intervals (CIs) and standard deviations for all metrics.

Model robustness was further validated through permutation tests, which confirmed chance-level performance when class labels were shuffled. Calibration curves were generated for the top-performing models, showing good agreement between predicted probabilities and observed outcomes. ROC and Precision–Recall curves with bootstrapped CIs were also reported, alongside confusion matrices for error analysis.

#### Gene set enrichment analysis of familial and sporadic MD

Gene Set Enrichment Analysis (GSEA) was performed on the top-ranked genes according to CADD and biallelic variant scores to identify potentially active pathways in both familial and sporadic MD (Tables S2, S3, S4, S5, and S6). KEGG and Gene Ontology (GO) enrichment analyses were conducted using clusterProfiler v.4.14.6,^34^ Reactome pathway analysis using ReactomePA v.1.50.0,^35^^,^^36^ and gene annotation was performed with org.Hs.e.g.,.db v.3.20.0. Gene Ontology enrichment was analyzed separately for Biological Process (BP), Cellular Component (CC), and Molecular Function (MF). All pathway analyses were filtered using a significance cutoff of *p* value < 0.05 with FDR correction.

### Quantification and statistical analysis

#### Feature ablation analysis

We conducted an ablation analysis to quantify the contribution of pre-specified feature blocks to the predictive performance of the final selected classifier (GB). For each ablation setting, we removed one feature group and re-executed the full model-selection protocol (training-set cross-validated tuning, threshold selection on the validation set using Youden’s J, and evaluation on a held-out test set). Performance was summarized using the following test-set metrics (AUC, AP, REC1, PREC1, F1, BAL_ACC, MCC, and BRIER), together with bootstrap 95% confidence intervals and deltas relative to the baseline model.

## Supplemental information

Document S1. Figures S1–S3

Table S1. Model evaluation, metrics, and notation

Table S2. Top 1,000 genes in familial MD according to the BVIS for each gene

Table S3. Top 1,000 genes in sporadic MD according to the BVIS for each gene

Table S4. Overlapping genes in familial and sporadic MD according to the BVIS

Table S5. Top 1,000 genes in FMD according to the CADD Phred score per gene

Table S6. Top 1,000 genes in sporadic MD according to the CADD Phred score per gene

Table S7. Cohorts used in the study to train, test, and evaluate the classifier
