# Supplementary material for: A machine learning classifier reveals hidden rare biallelic heterozygous variants in the coding regions of Meniere disease genome
Source: iScience. 2026 Oct 15;29(11):117838. doi: 10.1016/j.isci.2026.117838 (PMC13691498; doi:10.1016/j.isci.2026.117838)

iScience, Volume *29*

Supplemental information

A machine learning classifier reveals hidden rare biallelic heterozygous variants in the coding regions of Meniere disease genome

Sayedali A. Mohseni, Muhammad Hamza B. Khan, Pablo Cruz-Granados, Kiana Bagheri-Lotfabad, Amin Beheshti, and Jose A. Lopez-Escamez

Table of Contents

Supplementary Figure 1.- Models used to test MD classification. 2

Supplementary Figure 2.- Negative Control A (no training) and negative Control B (simulation) (A) Negative control analysis in which non-Meniere Disease (MD) samples were repeatedly assigned to two artificial groups of equal size, while keeping the trained model and predicted probabilities fixed. Across 1,000 random partitions, the difference in mean predicted probability between groups was centred at zero (mean ≈ −0.0003; 95% empirical interval −0.029 to 0.027). (B) Simulation-based negative control in which random 50/50 labels were assigned to the non-MD samples, followed by repeated cross-validated training of the gradient boosting pipeline. Across 200 trials, the resulting ROC AUC distribution was centred at chance level (mean AUC ≈ 0.50). 3

Supplementary Figure 3.- Non-MD Dataset. Distribution of predicted Meniere Disease (MD)–like probabilities obtained by applying the published gradient boosting model to a non-MD cohort (n = 101). As the dataset lacks ground-truth diagnostic labels for MD, standard discrimination metrics such as accuracy or ROC AUC are not applicable. Instead, this figure summarises the model's output scores in an unlabelled external cohort. All non-MD samples received predicted probabilities above the decision threshold used in the trained model, with scores concentrated between 0.68 and 0.73. 4

Supplementary Figure 1.- Models used to test MD classification.

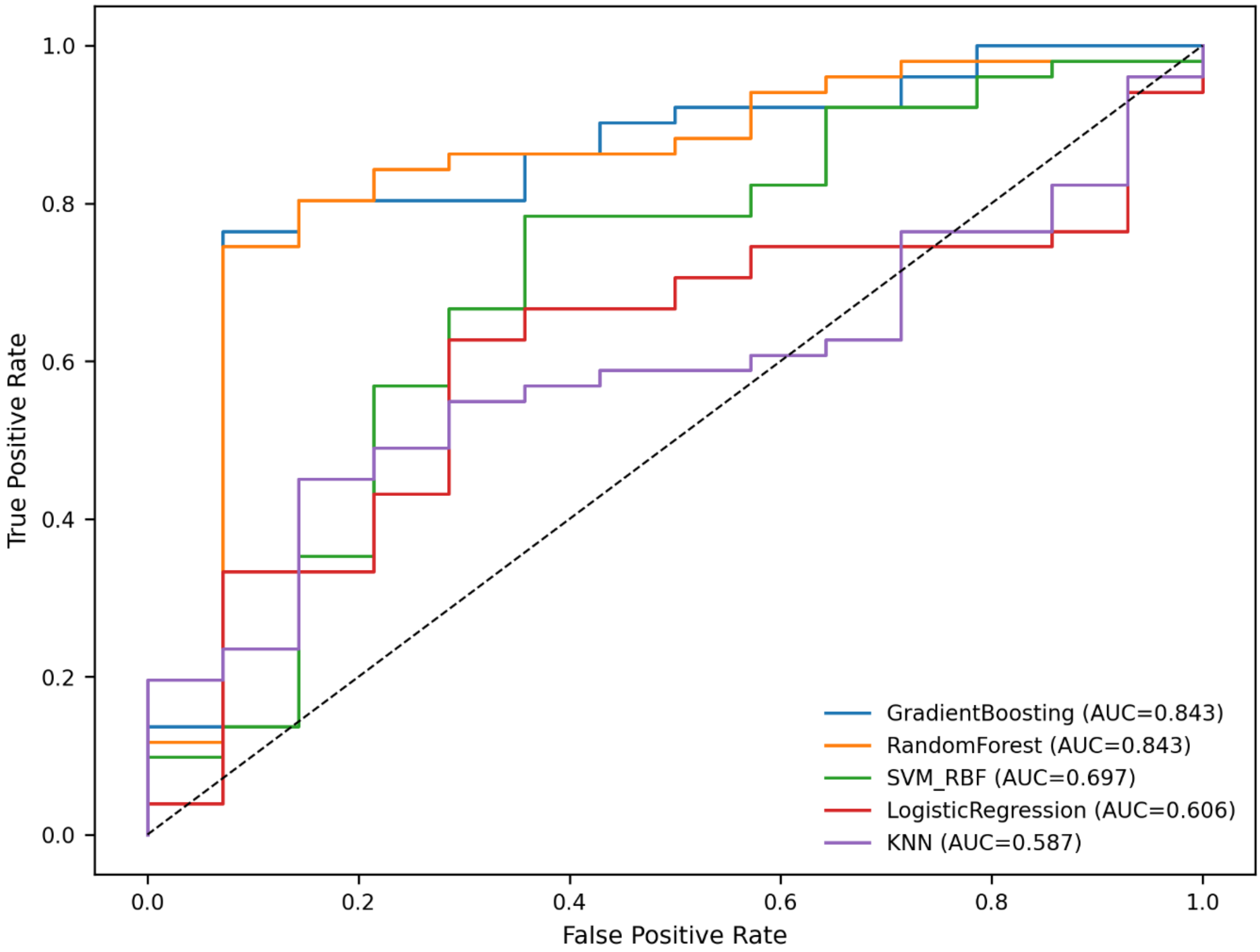

Supplementary Figure 2.- Negative Control A (no training) and negative Control B (simulation) (A) Negative control analysis in which non-Meniere Disease (MD) samples were repeatedly assigned to two artificial groups of equal size, while keeping the trained model and predicted probabilities fixed. Across 1,000 random partitions, the difference in mean predicted probability between groups was centred at zero (mean ≈ −0.0003; 95% empirical interval −0.029 to 0.027). (B) Simulation-based negative control in which random 50/50 labels were assigned to the non-MD samples, followed by repeated cross-validated training of the gradient boosting pipeline. Across 200 trials, the resulting ROC AUC distribution was centred at chance level (mean AUC ≈ 0.50).

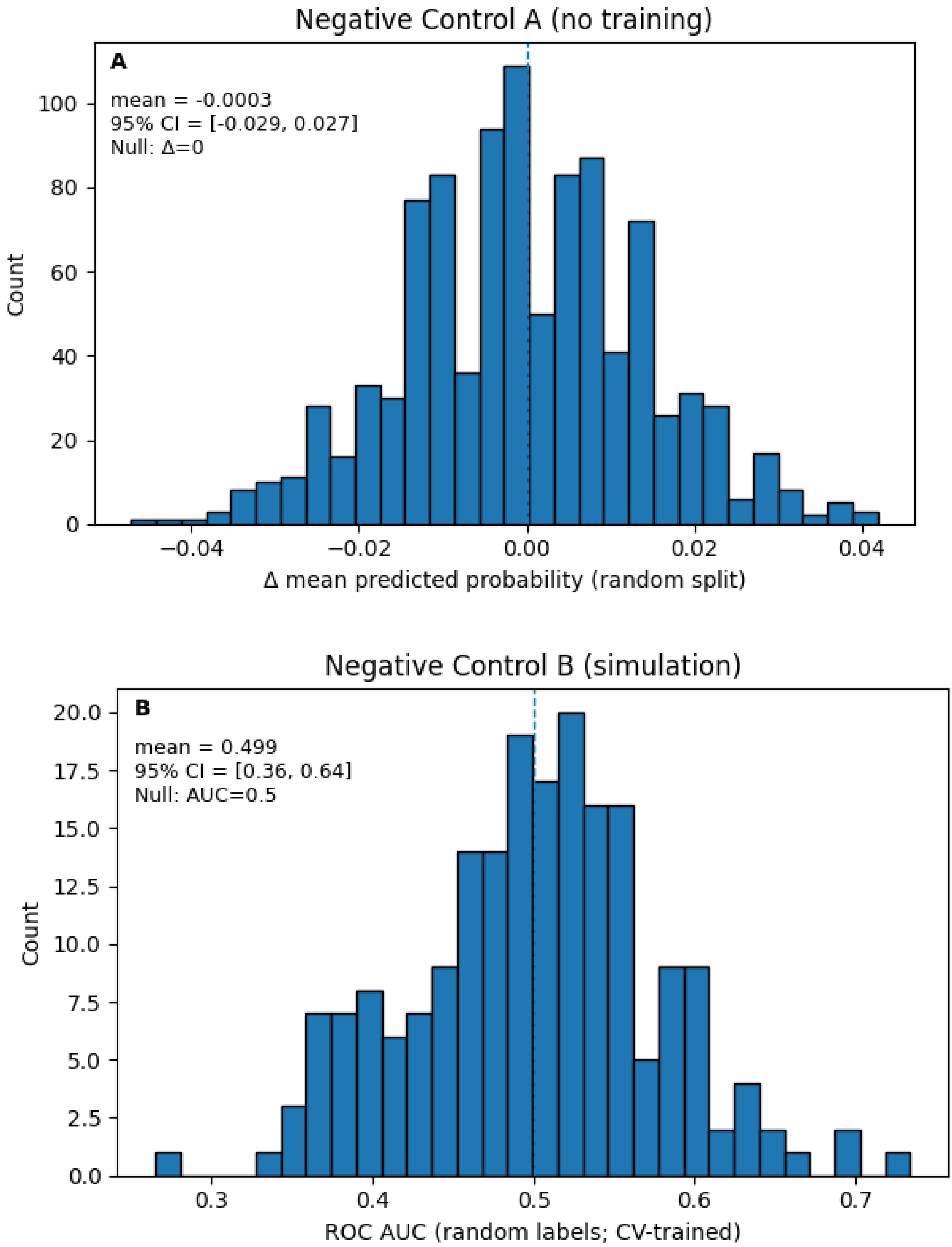

Supplementary Figure 3.- Non-MD Dataset. Distribution of predicted Meniere Disease (MD)–like probabilities obtained by applying the published gradient boosting model to a non-MD cohort (n = 101). As the dataset lacks ground-truth diagnostic labels for MD, standard discrimination metrics such as accuracy or ROC AUC are not applicable. Instead, this figure summarises the model's output scores in an unlabelled external cohort. All non-MD samples received predicted probabilities above the decision threshold used in the trained model, with scores concentrated between 0.68 and 0.73.

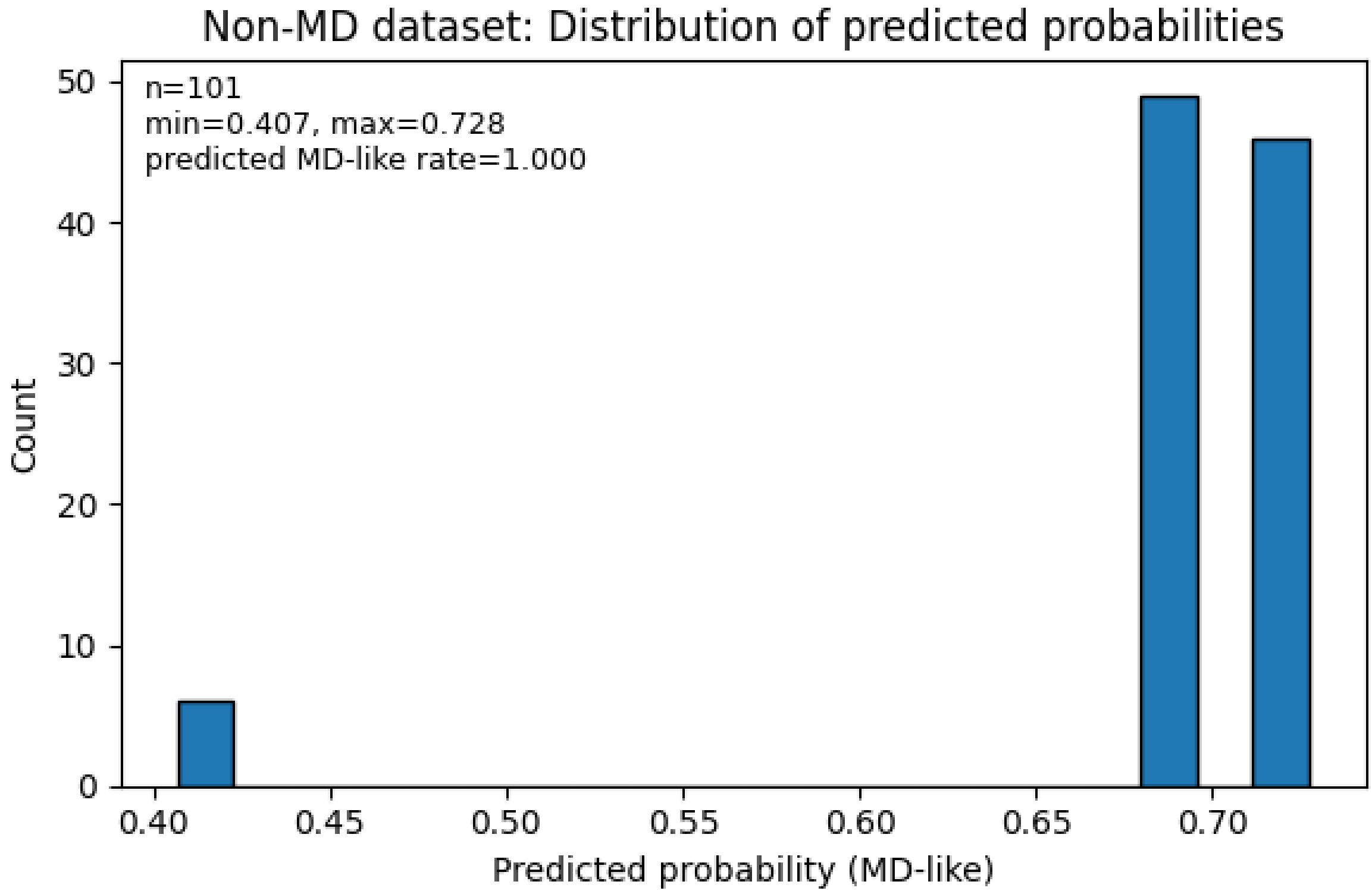

Supplement: Supplementary file 1 [file mmc1.pdf]
